# Blantyre Malaria Project Epilepsy Study (BMPES) of neurological outcomes in retinopathy-positive paediatric cerebral malaria survivors: a prospective cohort study

**DOI:** 10.1016/S1474-4422(10)70270-2

**Published:** 2010-12

**Authors:** Gretchen L Birbeck, Malcolm E Molyneux, Peter W Kaplan, Karl B Seydel, Yamikani F Chimalizeni, Kondwani Kawaza, Terrie E Taylor

**Affiliations:** a Michigan State University, International Neurologic and Psychiatric Epidemiology Program, East Lansing, MI, USA; b Malawi-Liverpool-Wellcome Trust Clinical Research Programme, College of Medicine, Malawi, and The Liverpool School of Tropical Medicine, University of Liverpool, Liverpool, UK; c Johns Hopkins Health Systems, Department of Neurology, Baltimore, MD, USA; d University of Malawi College of Medicine, Blantyre Malaria Project, Blantyre, Malawi; e Michigan State University, College of Osteopathic Medicine, East Lansing, MI, USA

## Abstract

**Background:**

Cerebral malaria, a disorder characterised by coma, parasitaemia, and no other evident cause of coma, is challenging to diagnose definitively in endemic regions that have high rates of asymptomatic parasitaemia and limited neurodiagnostic facilities. A recently described malaria retinopathy improves diagnostic specificity. We aimed to establish whether retinopathy-positive cerebral malaria is a risk factor for epilepsy or other neurodisabilities.

**Methods:**

Between 2005 and 2007, we did a prospective cohort study of survivors of cerebral malaria with malaria retinopathy in Blantyre, Malawi. Children with cerebral malaria were identified at the time of their index admission and age-matched to concurrently admitted children without coma or nervous system infection. Initially matching of cases to controls was 1:1 but, in 2006, enrolment criteria for cerebral malaria survivors were revised to limit inclusion to children with cerebral malaria and retinopathy on the basis of indirect ophthalmoscopic examination; matching was then changed to 1:2 and the revised inclusion criteria were applied retrospectively for children enrolled previously. Clinical assessments at discharge and standardised nurse-led follow-up every 3 months thereafter were done to identify children with new seizure disorders or other neurodisabilities. A Kaplan-Meier survival analysis was done for incident epilepsy.

**Findings:**

132 children with retinopathy-positive cerebral malaria and 264 age-matched, non-comatose controls were followed up for a median of 495 days (IQR 195–819). 12 of 132 cerebral malaria survivors developed epilepsy versus none of 264 controls (odds ratio [OR] undefined; p<0·0001). 28 of 121 cerebral malaria survivors developed new neurodisabilities, characterised by gross motor, sensory, or language deficits, compared with two of 253 controls (OR 37·8, 95% CI 8·8–161·8; p<0·0001). The risk factors for epilepsy in children with cerebral malaria were a higher maximum temperature (39·4°C [SD 1·2] *vs* 38·5°C [1·1]; p=0·01) and acute seizures (11/12 *vs* 76/120; OR 6·37, 95% CI 1·02–141·2), and male sex was a risk factor for new neurodisabilities (20/28 *vs* 38/93; OR 3·62, 1·44–9·06).

**Interpretation:**

Almost a third of retinopathy-positive cerebral malaria survivors developed epilepsy or other neurobehavioural sequelae. Neuroprotective clinical trials aimed at managing hyperpyrexia and optimising seizure control are warranted.

**Funding:**

US National Institutes of Health and Wellcome Trust.

## Introduction

Cerebral malaria, a disorder characterised by coma, parasitaemia, and no other evident cause of coma,^1^ affects about half a million children every year, primarily in sub-Saharan Africa.^2^ The case-fatality rate of cerebral malaria is estimated to be about 15%, with risk factors for death being deeper coma or coma of longer duration, younger age, seizures, hypoglycaemia, and hyperparasitaemia.^3^, ^4^ Neurological sequelae, including cortical blindness, gross motor deficits, ataxia, language regression, epilepsy, and behavioural abnormalities, have been reported at discharge in 10–20% of survivors, with follow-up assessments suggesting that gross deficits usually resolve within a few weeks of recovery.^4^, ^5^, ^6^, ^7^, ^8^, ^9^

Most previous studies of neurological sequelae related to cerebral malaria have been limited by poor retention rates, short duration of follow-up, and assessments that were stopped when the results of neurological examinations were normal. Delayed cerebral malaria sequelae, such as epilepsy, have been reported in studies designed to identify them: a retrospective cohort study in Kenya^10^ reported that 14 (9·2%) of 152 survivors developed epilepsy compared with four (2·2%) of 179 in the control group, and in Mali, a prospective study^11^ identified epilepsy in five (4·9%) of 101 survivors of cerebral malaria compared with one (0·5%) of 222 controls. Cognitive impairment has also been reported after recovery from cerebral malaria.^12^, ^13^, ^14^ However, these longer-term findings had diagnostic limitations. Cerebral malaria is challenging to diagnose definitively in endemic regions in which there are high rates of asymptomatic parasitaemia and limited neurodiagnostic resources. Autopsy studies show that about 23% of children meeting the standard clinical case definition of cerebral malaria have a non-malarial cause for coma and lack CNS sequestration of parasitised erythrocytes.^15^ Previous outcome studies, which are reviewed systematically in the webappendix, have relied upon the standard clinical case definition of cerebral malaria.

Malaria retinopathy, recently described in children, enhances the potential for diagnosis with 95% sensitivity and 90% specificity as compared with autopsy findings as the gold standard.^16^ Children meeting the standard clinical case definition of cerebral malaria who lack malaria retinopathy have a higher prevalence of pre-existing neurodisabilities and of family history of epilepsy compared with matched controls, which suggests that children with retinopathy-negative cerebral malaria have a pre-existing propensity toward adverse neurological symptoms and outcomes.^17^ Adverse neurological outcomes previously reported in cerebral malaria might have included children with neurological abnormalities preceding their malarial infection, and these pre-existing abnormalities might have contributed to the malaria-associated coma.

To establish whether retinopathy-positive cerebral malaria is a risk factor for epilepsy or other neurodisability and, if so, whether there are modifiable risk factors that offer potential opportunities for early intervention, we did a prospective cohort study of children with retinopathy-positive cerebral malaria. Follow-up assessments were designed to identify interim seizures and new neurodisabilities.

## Methods

### Participants

We did a prospective cohort study of cerebral malaria survivors who were admitted to the paediatric research ward of Queen Elizabeth Central Hospital (QECH), Blantyre, Malawi, during the malaria season (January–June) from 2005 to 2007 and who resided within the catchment area (within 1·5 h travel time from QECH by four-wheeled drive vehicle, with distances varying due to road conditions). Because QECH is in a region endemic for malaria, which is one of the commonest causes of illness in Malawian children, all comatose children admitted to QECH have a thick blood film reviewed to screen for *Plasmodium falciparum*. In Blantyre, about 83% of caregivers of children younger than 5 years cited fever and malaria as the impetus for seeking medical care in 2006–07.^18^ The group with cerebral malaria originally included all surviving children for whom consent was obtained and who met the standard clinical case definition of cerebral malaria: Blantyre coma score (BCS)^19^ less than or equal to 2, *P falciparum* parasitaemia, and no other evident cause of coma. We used the Epilepsy Screening Questionnaire to exclude children with a history of previous unprovoked seizures. This questionnaire has been previously used with 79·3% sensitivity and 92·9% specificity for detection of epilepsy.^20^ We applied the Ten Questions screen^21^, ^22^ to identify children with preadmission neurodisabilities. This screen detects neurodevelopmental disabilities in children and has been shown to be valid in children as young as 2 years in similar environments with good reliability (κ=0·67) and 85% sensitivity. Children with pre-existing neurodisabilities were eligible for enrolment and were followed up for the outcome of epilepsy, but were not included in analyses of subsequent neurodisabilities. There was no strict age limit and any comatose child who could fit in the paediatric cots on the ward was eligible for admission.

Throughout recruitment, all patients with cerebral malaria had an ophthalmological assessment on admission. In 2006–07, enrolment criteria for cerebral malaria survivors were revised to limit case inclusion to children with retinopathy-positive cerebral malaria on the basis of indirect ophthalmoscopic examination through a dilated pupil by a qualified ophthalmologist. Patients without malaria retinopathy and a small number of children who did not have a retinal examination while comatose were excluded from this analysis. These patients have been described elsewhere.^17^ The revised inclusion criteria used from 2006 onward were applied retrospectively to the children enrolled in 2005.

The control group consisted of the first eligible child who was admitted to the general paediatric service after each case was enrolled and who was age-matched to within 6 months. Inclusion criteria for age-matched controls were residence within 1·5 h travel from QECH by four-wheeled drive vehicle, a normal level of consciousness, and no history of unprovoked seizures before the index admission. Children with a brief febrile seizure before admission who were conscious on admission were eligible. Children with *P falciparum* parasitaemia were eligible as controls, although the presence of parasitaemia was not a requirement and not all control children were tested for malaria. The comparison group was also screened acutely with the Epilepsy Screening Questionnaire and the Ten Questions screen to identify children with a history of previous unprovoked seizures and those with pre-existing neurodisabilities. The findings from these questionnaires were used in the unexposed comparison group exactly as they were in the exposed group. Children with a history of unprovoked seizures were excluded from enrolment. Those with a history of neurodisability were eligible for inclusion but were excluded from analyses of the neurodisability outcome.

Our study was approved by the University of Malawi College of Medicine Research Ethics Committee and Michigan State University Biomedical Institutional Review Board. Signed informed consent was provided by the parents or guardians of the study participants.

### Procedures

We planned to recruit 225 cerebral malaria survivors matched 1:1 with unexposed controls. The addition of malaria retinopathy as an inclusion criterion in 2006 reduced the number of qualifying participants. Therefore, matching was increased to 1:2, to increase precision in estimates of differences between groups. Recruitment was continued through the 2007 malaria season with the goal of enrolling as many children as possible and assessments continued through to December, 2009, to allow at least 18 months of follow-up for the last children enrolled. Therefore, children enrolled earlier in the cohort were followed up for longer than children enrolled later.

For both cases and controls, several clinical characteristics, including established risk factors for seizures and epilepsy, were documented at enrolment. A research nurse interviewed caregivers (usually the mother) with the Epilepsy Screening Questionnaire and the Ten Questions screen, and recorded demographic data including age and sex. Potential pre-existing risk factors for epilepsy and adverse neurological outcomes were assessed including family history of epilepsy,^23^, ^24^ questions on delayed cry (was the child's birth problematic? did the child cry immediately after birth?),^25^ and birthweight from the health passport or under-5s card, if available.^26^ Family history was established with the Epilepsy Screening Questionnaire. If a parent or sibling screened positive, further follow-up questions were asked to confirm that this was a primary relative related to the patient biologically and to clarify the age of the person at the time they experienced the seizures. If the relative identified was not a primary relative, had experienced only a single seizure, or had seizures in association with childhood fever or illness, the child was not classified as having a family history of epilepsy. We also noted any previous history of severe malaria, defined as malaria that needed hospital admission.

On admission to the paediatric research ward, we recorded details of the general and neurological examination. We did not commence enrolment in the long-term follow-up study until survival was probable, usually on day 2 or 3 of the admission after acute data had already been collected. Laboratory investigations included full blood count, packed cell volume, lactate and glucose concentration, and thick and thin blood smears (with Field's stains) to identify the species and quantify malaria parasitaemia. Temperature and coma score were recorded serially. Seizures related to cerebral malaria were recorded on the basis of clinical identification as well as electroencephalogram (EEG) findings.

For patients with cerebral malaria, digital EEGs were obtained on the day of admission and daily thereafter until recovery from coma. EEGs were recorded with a BioLogic Ceegraph digital machine (Natus Medical Incorporated, San Carlos, CA USA) with a modified 10–20 system and met the American Electroencephalography Society guidelines for EEG.^27^ EEGs were interpreted acutely for clinical purposes and later categorised with the modified Synek classification system^28^, ^29^ (GLB). 36 (25%) of 146 EEGs had a second interpretation and modified Synek score assigned by an epileptologist (PWK) masked to everything except the BCS and patient age, to assess the validity of the initial acute interpretations and Synek scoring.

At discharge, neurological assessment was completed by the attending physician (a paediatrician or internist), and for all study participants discharge diagnoses and body-mass index were recorded. Children were then reviewed by a study nurse at 1 month after discharge and then every 3 months until the study was completed. At follow-up assessments, which were done at the hospital or via home-based visits led by nurses, study nurses repeated the Epilepsy Screening Questionnaire and the Ten Questions screen to identify any interim seizures or newly evident neurodisabilities. All children who were discharged with neurological sequelae, had interim seizures, or screened positive on the Ten Questions screen during follow-up were assessed by a neurologist (GLB) to characterise their seizure disorder or the nature of the neurological disability. Any additional concerns or issues from either the research nurses or the parents also triggered a physician-level review of the child. The examining neurologist and study nurses were all involved in the acute care of the study participants and thus exposure status was not masked. Children who initially screened negative on the Ten Questions screen and who later exhibited motor, sensory, or language impairment were deemed to have a new neurodisability. Interim seizures that happened during follow-up were deemed provoked seizures if health care was sought and a fever was documented, the parent reported a tactile temperature or measured a fever around the time of the seizure, the parent reported an illness preceding the seizure, or parasitaemia was recorded on the day of the seizure even in the absence of acute fever. More than one unprovoked seizure during follow-up was needed for a child to meet the diagnostic criteria for epilepsy. If a child's family moved outside of our defined catchment area, the child was no longer followed up unless the parents brought them to the paediatric research ward for their quarterly assessments.

Several children without seizures or neurodisability were referred for physician-level assessment because of parental concerns about new behavioural problems. Assessment of these children by a neurologist (GLB) in two different settings (at home and school or at their parental home and at a relative's home) were done and all met Diagnostic and Statistical Manual of Mental Disorders (4th edn) criteria for hyperactive-impulsive attention-deficit hyperactive disorder (ADHD).^30^ Although ADHD was not a prespecified outcome in our study, we include an analysis of this disruptive behavioural disorder group.

At the time of enrolment, neither acute neuroimaging nor ventilatory support was available on the QECH paediatric research ward. All children with cerebral malaria received treatment for acute severe malaria according to national guidelines that included blood transfusions if indicated, intravenous quinine, and benzodiazepines or paraldehyde for acute symptomatic seizures. For refractory seizures, additional antiepileptic drugs, usually phenobarbital, were given.

### Statistical analysis

Recruitment of 225 cerebral malaria survivors matched 1:1 with unexposed controls would have provided 87% power to detect an 8% difference in epilepsy development. Data were originally recorded on paper forms with subsequent double data entry into Microsoft Office Excel 97–2003 software. This was then imported into EPI INFO (version 3.5) for analysis. Calculations of geometric means for parasitaemia were completed using Dimensions Research online software.^31^

The admission characteristics of retinopathy-positive cerebral malaria survivors and controls were compared with *t* tests and odds ratios (ORs). Owing to the revised inclusion criteria in 2006–07 for cerebral malaria cases, and to use data obtained from all controls, we did an unmatched analysis comparing the group of cerebral malaria survivors versus controls. We assessed the ORs of incident epilepsy, new neurodisability, disruptive behavioural disorders, any adverse neurological outcome, and death. We did a Kaplan-Meier survival analysis for incident epilepsy, our primary outcome of interest. Any children lost to follow-up were censored from the data on the day after their last follow-up. To identify potential risk factors for adverse neurological outcomes in cerebral malaria survivors, we also assessed acute clinical characteristics associated with adverse neurological outcomes (epilepsy, neurodisability, disruptive behavioural disorder, or the pooled outcome of any adverse neurological sequelae) with the *t* test or OR with 95% CIs. For any χ^2^ assessment with less than five items of data in a cell, Fisher's exact test was used. For *t*-test comparisons, Bartlett's test for population variance was assessed and where indicated (ie, if there was significant population variance [p<0·05]) the Kruskal-Wallis (non-parametric) test was used. Because of the variance from our initial recruitment and analysis plan, we also did a sensitivity analysis of the risk of each adverse neurological outcome with matched pairs that included the first matched control selected for each case.

### Role of the funding source

The sponsor of the study had no role in study design, data collection, data analysis, data interpretation, or writing of the report. The corresponding author had full access to all the data in the study and had final responsibility for the decision to submit for publication.

## Results

**Figure 1:**
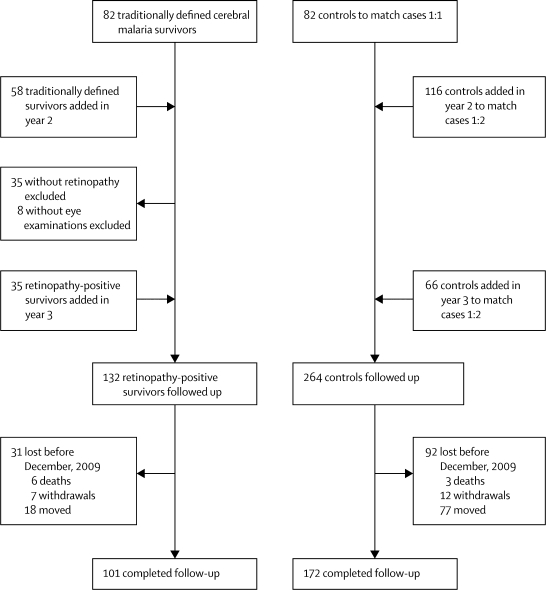
Cohort recruitment

Figure 1 shows cohort recruitment. 132 children with retinopathy-positive malaria and 264 controls were recruited and followed up for a mean of 544 days (median 495; IQR 195–819). The most common discharge diagnoses in the control group were malaria, pneumonia, and gastroenteritis, accounting for 190 controls. Children in the control group who had malaria were admitted because of a febrile seizure at home, anaemia, or possible anaemia—children with uncomplicated malaria are generally not admitted.

The duration of follow-up from recruitment to an adverse outcome or attrition did not differ significantly between cases and controls (mean 534 days [SD 453] *vs* 446 [374]; p=0·13). However, follow-up every 3 months was maintained through to December, 2009, in 101 (77%) of 132 cases and 172 (65%) of 264 controls (p=0·02). Attrition was due to death in six cases (5%) and three controls (1%), to withdrawal in seven cases (6%) and 12 controls (5%), and to family relocation outside of the tracking area in 18 cases (14%) and 77 controls (29%).

We provided ongoing medical care to the 42 children who had experienced adverse neurological outcomes. Among these children, 14 had families who relocated outside of the tracking area during this study, but parents for all 14 of these children continued to bring them in for assessments. Had these children been withdrawn from the study at the time of the relocation, the rate of study completion in the cerebral malaria survivors would have been 53% (70 of 132). To our knowledge, there were no children in the control group whose parents continued to bring them for quarterly assessments after family relocation outside the tracking area. When we compared the age, sex, enrolment date, and outcome of children who completed follow-up through to December, 2009, with those of children who did not complete follow-up, more recent enrolment and having an adverse neurological outcome were both associated with higher completion rates. There was no difference in the proportion of cases versus controls who continued follow-up for the full duration of the study once the children with adverse neurological outcomes were excluded from the comparison (60 [67%] of 90 cases *vs* 170 [65%] of 261 controls; p=0·48).

Table 1 shows the characteristics of the cerebral malaria cases and controls at admission. Inter-rater agreement in ratings from the two independent EEG readers (GLB and PWK) for the eight-category modified Synek classification system was 0·88 (95% CI 0·78–0·98), with no disagreement on electrographic seizure identification. Nine (19%) of 48 children who had no witnessed seizures before admission and no clinical evidence of seizure on admission had seizure activity evident on their admission EEG.

**Table 1:** Characteristics at admission

	**Cerebral malaria survivors (n=132)**	**Controls (n=264)**	**Odds ratio (95% CI)**
Male	65 (49%)	148 (56%)	0·76 (0·50–1·16)
Age (months)	42·3 (25·1)	42·8 (27·5)	1·00 (0·99–1·01)
Body-mass index (kg/m^2^)	15·6 (2·9)	15·6 (3·0)	1·00 (0·93–1·07)
Axillary temperature on admission (°C)	38·3 (1·3)	38·4 (1·2)	0·94 (0·79–1·11)
Birthweight (kg)*	3·2 (0·6)	3·2 (0·7)	1·63 (0·55–4·78)
Problem birth or delayed cry	8 (6%)	13 (5%)	1·25 (0·50–3·08)
Pre-existing neurodisability†	11 (8%)	11 (4%)	2·09 (0·88–4·96)
History of severe malaria‡	6 (5%)	12 (5%)	1·00 (0·37–2·73)
Family history of epilepsy	10 (8%)	12 (5%)	1·72 (0·72–4·09)

Data are n (%), odds ratio (95% CI), or mean (SD).

* 62 cerebral malaria survivors and 173 controls.

† Established through the Ten Questions screen.

‡ Defined as a malaria infection that needed hospital admission.

**Figure 2:**
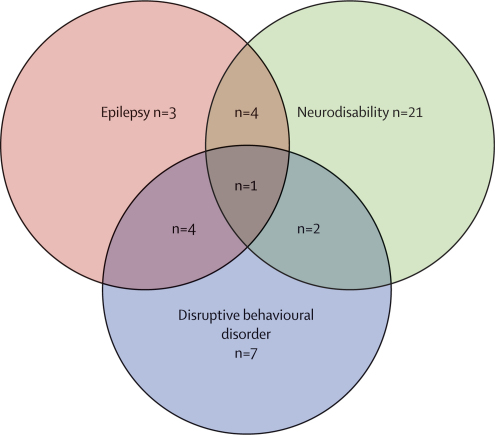
Distribution of comorbid neurological sequelae after cerebral malaria

Table 2 shows outcomes in cerebral malaria survivors versus unexposed controls. Cerebral malaria survivors were at raised risk of epilepsy, new neurodisabilities, and disruptive behavioural disorders. Overall, 42 cerebral malaria survivors (32%) had at least one adverse neurological outcome. The case-fatality rate during follow-up was greater for cerebral malaria survivors than for controls (table 2). These findings remained significant in the matched sensitivity analysis (data not shown). Figure 2 shows the distribution and comorbidity of neurological sequelae among the cerebral malaria survivors. Multiple adverse neurological outcomes happened sequentially, with motor, sensory, or language deficits being evident initially (median 111 days after discharge, IQR 23–228), then disruptive behavioural disorders (150 days, 83–228), and lastly epilepsy (309 days, 111–524). The time to adverse outcome is for all patients, not just those with more than one event.

**Table 2:** Outcomes in cerebral malaria survivors and controls

	**Cerebral malaria survivors**	**Controls**	**Odds ratio (95% CI)**
Epilepsy	12/132 (9%)	0/264 (0%)	Undefined (4·8–15·3 [cases]; 0–1·4 [controls])
Disruptive behavioural disorder	14/132 (10·6%)	1/264 (0·4%)	31·2 (4·1–240·1)
New neurodisabilities*	28/121 (23·1%)	2/253 (0·8%)	37·8 (8·8–161·8)
Death	6/132 (4·5%)	3/264 (1·1%)	4·1 (1·02–16·8)

Outcomes are not mutually exclusive. Data are n/N (%) or OR (95% CI).

* Excludes children with a history of neurodisability at the time of presentation.

**Figure 3:**
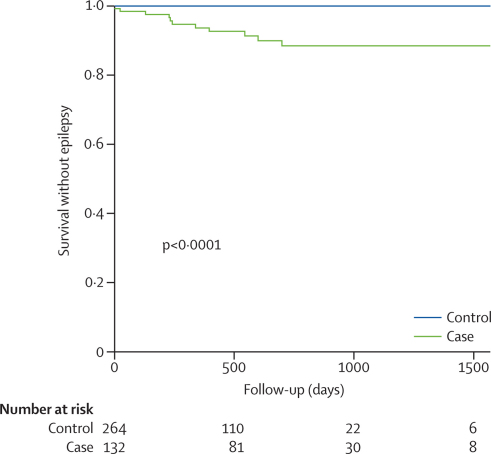
Kaplan-Meier curve for epilepsy outcome Statistical significance is by log-rank test.

Figure 3 shows the Kaplan-Meier survival analysis for incident epilepsy in cerebral malaria survivors versus controls. Epilepsy in cerebral malaria survivors was characterised by localisation-related epilepsy with the epileptic focus (established by seizure semiology, EEG, and neuroimaging) corresponding to focal brain regions involved with status epilepticus or repeated seizures during the acute cerebral malaria infection.^32^ The children who developed epilepsy all needed treatment for recurrent seizures, many had refractory seizures that needed more than one antiepileptic drug, and two died in status epilepticus. Table 3 provides further details of the epilepsy syndrome after cerebral malaria.

**Table 3:** Clinical characteristics of epilepsy after cerebral malaria

	**Age at index admission (months)**	**Seizure types**	**Seizure frequency**	**Treatment**	**Clinical course**
Child 1	12	Focal motor with secondary generalisation	Two to three per month on treatment	Phenobarbital	Mother concerned that two antiepileptic drugs caused worsening in already compromised gait and unwilling to use second antiepileptic drug
Child 2	32	Generalised tonic clonic	Seizure free on treatment	Carbamazepine	Poor response to phenobarbital, which caused behavioural problems and aggression
Child 3	50	Generalised tonic clonic	One to two per month without treatment	None	Tried phenobarbital, then carbamazepine. Both decreased seizure frequency but were associated with aggressive behaviours so parents declined further treatment
Child 4	10	Right upper-extremity focal motor with or without secondary generalised tonic clonic	Seizure free on treatment	Phenobarbital	Mother reported breakthrough seizures whenever they ran out of medication for more than 1 week
Child 5	23	Generalised tonic clonic	Two to three per week off treatment	Phenobarbital, then carbamazepine, then both	Decreased seizure frequency to about one per week and decreased duration to minutes
Child 6	11	Focal motor and generalised tonic clonic	Multifocal and generalised tonic clonic daily	Phenobarbital with carbamazepine	Spastic quadriparesis, non-verbal, frequent seizures. Died at home of status epilepticus 2 weeks after family ran out of phenobarbital
Child 7	37	Generalised tonic clonic	One per month on average; more with any febrile illness	Phenobarbital	Limited follow-up details available
Child 8	28	Generalised tonic clonic and possible myoclonic	Two to three per week (brief and in clusters)	Phenobarbital with carbamazepine	Spastic quadriparesis, non-verbal. Effect on quality of life unclear
Child 9	38	Multifocal with or without secondary generalised tonic clonic	Right focal motor initially	Phenobarbital	Controlled on phenobarbital. Last visit, mother requested that the child be weaned off antiepileptic drugs
Child 10	48	Complex partial seizures with secondary generalisation and post-ictal psychosis and aggression	One to two per month but many more with any febrile illness	Phenobarbital, then carbamazepine	Shorter duration of seizures when on carbamazepine. One admission to hospital other than QECH with status epilepticus associated with a fever from respiratory illness
Child 11	47	Generalised tonic clonic	Unclear—died before pattern could be fully assessed	Phenobarbital	Developed gastroenteritis and was treated at home despite severe dehydration. On day 4 of illness, developed status epilepticus and died on the way to hospital
Child 12	42	Generalised tonic clonic	Less than one per month, although clearly recurrent and more frequent with any febrile illness	Phenobarbital	Well controlled and mother interested in weaning from antiepileptic drugs

QECH=Queen Elizabeth Central Hospital.

At discharge, the attending physician identified hemiparesis or monoparesis in seven of 132 patients with cerebral malaria, hypotonia in four, gait ataxia in two, speech regression in four, and choreiform movements in one. These neurological deficits at discharge are consistent with previous studies of paediatric cerebral malaria sequelae at discharge from QECH.^33^ An additional 16 children had new neurodisabilities identified during follow-up. All 42 children with adverse neurological outcomes were examined by a neurologist: 12 had spastic quadriparesis usually accompanied by language regression or cortical blindness; 12 had isolated language regression or delayed speech; six had motor and language regression or delay; five had hemiparesis; four had monoparesis; and three had isolated ataxia. Outcomes were not mutually exclusive.

Table 4 lists potential risk factors for adverse neurological outcomes of cerebral malaria survivors. A higher maximum temperature was associated with epilepsy and disruptive behavioural disorders, a BCS on admission of 0 or 1 was associated with disruptive behavioural disorders, seizures during admission were a risk factor for epilepsy, and male sex was a risk for subsequent neurodisabilities.

**Table 4:** Risk factors for adverse neurological outcomes

	**Epilepsy (n=12)**	**Disruptive behavioural disorder (n=14)**	**Neurodisabilities (n=28)**	**Any adverse neurological outcome (n=42)**
Age (months)	41·3 (28·4) *vs* 42·4 (24·8); p=0·88	40·3 (28·8) *vs* 42·5 (24·7); p=0·75	49·3 (25·1) *vs* 39·9 (22·2); p=0·06	43·9 (23·9) *vs* 41·6 (25·7); p=0·62
Male	7/12 (58%) *vs* 58/120 (48%) OR 1·50 (0·45–4·98)	8/14 (57%) *vs* 57/118 (48%) OR 1·43 (0·47–4·37)	20/28 (71%) *vs* 38/93 (41%) OR 3·62 (1·44–9·06)*	6/41 (63%) *vs* 39/91 (43%) OR 2·31 (1·08–4·94)
Body-mass index (kg/m^2^)	17·0 (3·23) *vs* 15·5 (2·86); p=0·09	15·8 (3·79) *vs* 15·6 (2·81); p=0·78	15·8 (2·51) *vs* 15·3 (2·80); p=0·40	15·8 (2·93) *vs* 15·5 (2·92); p=0·58
Family history of epilepsy	1/12 (8%) *vs* 9/120 (8%) OR 1·12 (0·13–9·69)	1/14 (7%) *vs* 9/118 (8%) OR 0·93 (0·11–7·95)	4/28 (14%) *vs* 5/93 (5%) OR 2·93 (0·73–11·78)	5/36 (12%) *vs* 5/91 (6%) OR 2·39 (0·65–8·76)
Admission temperature (°C)	38·2 (1·25) *vs* 38·9 (1·36); p=0·08	38·4 (1·45) *vs* 38·3 (1·26); p=0·76	38·3 (1·34) *vs* 38·3 (1·28); p=0·98	38·4 (1·35) *vs* 38·2 (1·24); p=0·43
Blantyre coma score <2 at admission	8/12 (67%) *vs* 54/120 (45%) OR 2·44 (0·70–8·56)	11/14 (79%) *vs* 51/118 (43%) OR 4·82 (1·28–18·17)*	17/28 (61%) *vs* 40/93 (43%) OR 2·04 (0·89–4·96)	29/43 (67%) *vs* 33/89 (37%) OR 3·52 (1·63–7·59)
Haematocrit at admission (% packed cell volume per μL)	18·2 (5·02) *vs* 18·2 (5·49); p=0·98	18·4 (4·01) *vs* 18·2 (5·60); p=0·90	18·8 (7·35) *vs* 18·0 (4·95); p=0·86	18·6 (6·36) *vs* 18·0 (4·98); p=0·63
White blood cell count at admission (mean count per μL)	10 718 (3035) *vs* 14 010 (11 330); p=0·69	10 583 (3910) *vs* 14 057 (11 342); p=0·47	14 426 (6480) *vs* 13 480 (11 337); p=0·07	13 146 (5953) *vs* 13 935 (12 427); p=0·19
Positive blood culture	1/11 (10%) *vs* 11/114 (9%) OR 1·07 (0·12–9·15)	1/12 (8%) *vs* 11/113 (10%) OR 1·19 (0·14–10·08)	3/27 (9%) *vs* 8/88 (9%) OR 0·80 (0·20–3·25)	4/12 (9%) *vs* 78/113 (10%) OR 0·90 (0·25–3·18)
Parasitaemia (geometric mean parasite per μL)	18 138 (371 686) *vs* 25 388 (180 994); p=0·10	21 292 (371 385) *vs* 10 084 (227 806); p=0·09	18 491 (381 325) *vs* 25 345 (244 726); p=0·11	19 593 (395 388) *vs* 19 821 (250 958); p=0·93
Lactate >5 (mg/μL)	7/12 (58%) *vs* 62/115 (54%) OR 1·20 (0·36–3·99)	5/13 (39%) *vs* 64/114 (53%) OR 0·49 (0·15–1·58)	16/27 (59%) *vs* 48/91 (53%) OR 1·30 (0·54–3·11)	22/40 (55%) *vs* 47/87 (54%) OR 1·54 (0·74–3·24)
Hypoglycaemia†	6/12 (50%) *vs* 55/120 (46%) OR 1·39 (0·91–1·13)	6/14 (43%) *vs* 55/118 (47%) OR 0·98 (0·88–1·11)	16/28 (53%) *vs* 42/93 (44%) OR 1·09 (0·90–1·31)	22/41 (4%) *vs* 39/91 (43%) OR 1·10 (0·87–1·40)
Maximum temperature (°C)	39·4 (1·17) *vs* 38·5 (1·10); p=0·01*	39·2 (1·00) *vs* 38·6 (1·13); p=0·04*	38·6 (1·18) *vs* 38·6 (1·14); p=0·97	38·9 (1·17) *vs* 38·5 (1·10); p=0·08
Seizures during cerebral malaria‡	11/12 (92%) *vs* 76/120 (63%) OR 6·37 (1·02–141·20)*	9/14 (64%) *vs* 78/118 (66%) OR 0·92 (0·29–2·94)	20/28 (71%) *vs* 57/93 (61%) OR 1·58 (0·63–3·96)	30/41 (73%) *vs* 57/91 (3%) OR 1·63 (0·72–3·66)
Time to coma resolution (h)	46·6 (37·9) *vs* 53·4 (39·5); p=0·66	44·7 (22·1) *vs* 54·0 (40·9); p=0·70	48·3 (29·7) *vs* 53·9 (41·6); p=0·63	46·4 (25·5) *vs* 55·2 (43·0); p=0·61

Data are mean (SD), n/N (%), or OR (95% CI), for event versus no event. Outcomes are not mutually exclusive. OR=odds ratio.

* Significant findings.

† Defined as a glucose <2·2 mmol/L.

‡ Seizures related to cerebral malaria were documented on the basis of clinical identification as well as EEG findings.

## Discussion

Our prospective study describes neurological outcomes for a population of well characterised paediatric cerebral malaria survivors. Our acute clinical data provide information on the clinical characteristics predictive of brain injury in survivors (panel). Acute and serial EEGs were especially important for identifying subclinical seizures, and inclusion of cerebral malaria retinopathy in the diagnostic criteria improved the certainty of diagnosis. Previous cerebral malaria outcomes studies did not include retinal assessments and thus probably included children with pre-existing neurological injuries, a predisposition to adverse neurological outcomes, or a non-malarial cause of coma. The population studied and their outcomes are probably representative of children with cerebral malaria who receive care in tertiary care settings within sub-Saharan Africa, but might differ from children with cerebral malaria treated within more community-based settings, where disease might be less severe but care might be less assiduous.

Panel **Research in context**

**Systematic review**

A PubMed search with the keywords “epilepsy and malaria” and “malaria and neurologic sequelae” yielded 295 articles. 13 studies remained after elimination of studies of adults, case reports, and those with no information on neurological outcomes after discharge. None of these studies included malaria retinopathy in the diagnostic criteria.

**Interpretation**

The Blantyre Malaria Project Epilepsy Study (BMPES) provides prospective longitudinal outcome data on a population of patients with retinopathy-positive paediatric cerebral malaria. Findings show that cerebral malaria is a risk factor for subsequent epilepsy, disruptive behavioural disorders, and other neurodisabilities. The epilepsy syndrome that comes after cerebral malaria seems to be associated with localisation-related, often refractory, epilepsy. The risk factor analysis provides information on potential neuroprotective interventions, which include improved acute seizure control during cerebral malaria and more aggressive fever management.

Several limitations of our study deserve a mention. Overall, cases were followed up longer than controls. A longer follow-up and higher retention rate among cases might have caused us to overestimate the effects of cerebral malaria. However, the duration of follow-up from recruitment to initial adverse outcome or attrition did not differ significantly between cases and unexposed controls, and when children with adverse neurological outcomes were excluded from the comparison, the proportion of cases versus controls who continued follow-up for the full duration of the study did not differ. These findings suggest that the additional medical services provided to children with adverse neurological outcomes represented an incentive for ongoing follow-up visits, and confounding is probably limited.

We used the Epilepsy Screening Questionnaire to exclude children with a history of unprovoked seizures; however, it has not been validated for use in Malawian children. We used the Ten Questions screen to assess neurodisabilities. This screen lacks sensitivity in children younger than 2 years; 79 children (20%) in our study were younger than 2 years at the time of their first follow-up. We are not aware of any screening methods for identifying neurodisabilities in children younger than 2 years that have been validated for use by non-physician health-care workers in an African setting. Owing to age-based matching, extent of use of the Ten Questions screen in children younger than 2 years did not differ for cases versus controls and 382 (97%) of 396 children were screened at least once after reaching the age of 2 years.

Before we started our study in 2005, behavioural disorders had not been reported as sequelae of cerebral malaria and behavioural disorders were not a pre-specified outcome. Recent retrospective clinic-based studies from Mali^8^ and Uganda^9^ have reported behavioural problems in cerebral malaria survivors, corroborating our unexpected findings. Children assessed explicitly for behavioural problems in our study were only those brought to the attention of the research team by the troubled parents. The prevalence of disruptive behavioural disorders in Malawian children is not known, but the worldwide prevalence of ADHD is estimated to be 5·3%.^34^ No culturally validated methods yet exist for diagnosing ADHD or other disruptive behavioural disorders in Malawi. Further studies of behavioural problems after cerebral malaria are needed and will require the development and validation of appropriate instruments for characterising paediatric behavioural problems in this population. Longer term and more sophisticated assessments of epilepsy and of cognitive and behavioural outcomes are also needed.

Our findings show that retinopathy-positive cerebral malaria is a risk factor for several adverse neurological outcomes including epilepsy, disruptive behavioural disorders, and neurodisabilities characterised by motor, sensory, or language deficits. Most sequelae were delayed in presentation and hence were not evident at the time of discharge from the hospital after the initial illness. Cerebral sequestration of parasites is the hallmark of cerebral malaria and the only methods available for confirming CNS sequestration require analysis of brain tissue. As such, it is difficult to know how representative this population of children with retinopathy-positive cerebral malaria is of all children with cerebral malaria. If the findings of our study are generalisable, then about 135 000 African children younger than 5 years have neurological sequelae due to cerebral malaria-induced brain injury each year and cerebral malaria might be one of the more common causes of epilepsy in malaria-endemic regions.

The syndrome of epilepsy after cerebral malaria described in our cohort of cerebral malaria survivors is primarily localisation-related epilepsy that is often associated with other neurodisabilities and refractory to treatment with a single drug. Early mortality in two of 12 children who developed epilepsy suggests that retrospective studies might not identify epilepsy that results in early deaths.

The finding that boys were at greater risk for new neurodisability was unexpected and might suggest that the Ten Questions screen has some sex-based bias in the Malawi population. However, recent epidemiological studies have established that boys experience higher stroke mortality rates across all paediatric age ranges^35^ and also seem to be more susceptible to arterial, neonatal, traumatic, and non-traumatic strokes than are girls.^36^

Most of the risk factors for adverse neurological outcomes after cerebral malaria identified in this study are consistent with data from other causes of CNS injury. Hyperthermia is a risk factor for adverse outcome in stroke and traumatic brain injury,^37^, ^38^ and secondary ADHD has been reported after paediatric traumatic brain injury with prolonged coma.^39^ The relation in our study between acute seizures and later epilepsy might suggest an underlying causal pathway in which acute seizures induce epileptogenesis.^40^ Alternatively, such acute symptomatic seizures might simply suggest a completed, irrevocable, brain injury that predisposes to later epilepsy. Given the chronic burden of neurological disability and substantial loss of human capacity related to brain injury caused by cerebral malaria, neuroprotective clinical trials aimed at maintaining normothermia and improved acute seizure control are warranted. In previous clinical trials, phenobarbital used for aggressive seizure control in paediatric cerebral malaria was associated with increased mortality probably due to respiratory failure in the absence of mechanical ventilatory capacity.^41^ As such, future studies aimed at seizure control should use drugs that are less likely to cause respiratory suppression, such as levetiracetam.

## Web Extra Material

Supplementary webappendix
