# Supplementary material for: Blantyre Malaria Project Epilepsy Study (BMPES) of neurological outcomes in retinopathy-positive paediatric cerebral malaria survivors: a prospective cohort study
Source: Lancet Neurol. 2010 Dec;9(12):1173–81. doi: 10.1016/S1474-4422(10)70270-2 (PMC2988225; doi:10.1016/S1474-4422(10)70270-2)
Supplement: Supplementary webappendix [file mmc1.pdf]

THE LANCET Neurology

Supplementary webappendix

This webappendix formed part of the original submission and has been peer reviewed. We post it as supplied by the authors.

Supplement to: Birbeck GL, Molyneux ME, Kaplan PW, et al. Blantyre Malaria Project Epilepsy Study (BMPES) of neurological outcomes in retinopathy-positive paediatric cerebral malaria survivors: a prospective cohort study. *Lancet Neurol* 2010; published online Nov 4. DOI:10.1016/S1474-4422(10)70270-2.

A Systematic Review of Neurologic Sequelae following Cerebral Malaria

A PubMed search using two keywords "epilepsy AND malaria" and "malaria AND neurologic sequelae" yielded 295 articles. Studies involving only adults, case reports and those with no information on neurologic outcome after discharge were excluded. Retrospective studies were included, even if there was no clear sampling frame. None of these studies included cerebral malaria retinopathy in the diagnostic criteria.

Study	Number of subjects[1]	Follow-up Period	Neurologic sequelae at discharge (%)	Sequelae at follow-up (%)	Sequelae	Predictors of Sequelae
Carme et al. 1993 [1]	58	9-27 months	9	50	Cortical Blindness<br>Hypotonia<br>Ataxia	Young age<br>Depth of coma
Meremikwa et al 1997 [2]	39	3 weeks-3 years	28	27	Cortical blindness<br>Speech disorders<br>Motor dysfunction	Prolonged coma<br>Focal Seizures<br>Abnormal postures
Bondi 1992[3]	62	12-16	18	55	Monoparesis	Severe seizures

[1] Survivors of the acute cerebral malaria episode

		months			Cortical blindness<br>Speech disorders	Hypoglycemia
Brewster et al 1990 [4]	265	1-6 months	12	25	Hemiparesis<br>Cortical blindness<br>Aphasia<br>Ataxia	Biphasic course<br>Coma<br>Anemia<br>Seizures
Van Hensbroek et al 1997 [5]	418	1-6 months	23	4	Paresis<br>Ataxia<br>Hearing problems<br>Visual problems<br>Cognitive dysfunction	Depth of coma<br>Multiple seizures<br>Prolonged Coma<br>High temperature<br>Leukocytosis
Crawley et al 1996 [6]	58	1 month	7	0	Hemiparesis<br>Spastic gait<br>Cognitive dysfunction<br>Speech abnormalities	Young age<br>Status epilepticus

					Epilepsy	
Molyneux et al 1987 [7]	111	2 weeks-1 month	9	64	Hypotonia<br>Hemiparesis<br>Ataxia<br>Tremor	Seizures<br>Depth of coma<br>Young age<br>Hypoglycemia
Carter et al 2006[8-10]	152	Varied (months)	n/a	11.8% Language disorders<br>24% neurocognitive impairments<br>9.2% epilepsy		---------------------------
Boivin et al 2007[11]	44	6 months	n/a	21.4	Cognitive impairment (focused assessment)	Longer duration of coma<br>Seizures
Ngoungou et al 2007[12-14]	101	Months (varied)	7.9	19.8	Mental retardation<br>Speech delay<br>Frontal syndromes<br>Epilepsy ADHD<br>Hemiparesis	Family history of epilepsy<br>Febrile seizure

					Movement disorders	
Idro et al 2006[15]	143	≥ 20 months	23.8%	23.8%	Motor, speech/language, cognitive	Seizures, deep coma, focal deficits, age < 3 years, hypoglycemia
Idro et al 2010[16]	30 (retrospective from subspecialty clinic)	Varied (months)	Not available	85.2%	Motor deficits<br>Speech regression<br>hearing loss<br>Behavioral problems<br>Epilepsy<br>Blindness<br>Cognitive impairment	Hyporeflexia on admission and focal neurologic deficits at 3 months post CM
John et al[17]	44	24 months	----------	26.3%	Cognitive impairment	----------------------------

Bibliography

1. Carme B, Bouquety JC, Plassart H. Mortality and sequelae due to cerebral malaria in African children in Brazzaville, Congo. *Am J Trop Med Hyg* 1993;**48**(2)**:**216-21.

2. Meremikwu MM, Asindi AA, Ezedinachi E. The pattern of neurological sequelae of childhood cerebral malaria among survivors in Calabar, Nigeria. *Cent Afr J Med* 1997;**43**(8)**:**231-4.

3. Bondi FS. The incidence and outcome of neurological abnormalities in childhood cerebral malaria: a long-term follow-up of 62 survivors. *Trans R Soc Trop Med Hyg* 1992;**86**(1)**:**17-9.

4. Brewster DR, Kwiatkowski D, White NJ. Neurological sequelae of cerebral malaria in children. *Lancet* 1990;**336**(8722)**:**1039-43.

5. van Hensbroek MB, Palmer A, Jaffar S, Schneider G, Kwiatkowski D. Residual neurologic sequelae after childhood cerebral malaria. *J Pediatr* 1997;**131**(1 Pt 1)**:**125-9.

6. Crawley J, Smith S, Kirkham F, Muthinji P, Waruiru C, Marsh K. Seizures and status epilepticus in childhood cerebral malaria. *Q J Med* 1996;**89**(8)**:**591-7.

7. Molyneux ME, Taylor TE, Wirima JJ, Borgstein A. Clinical features and prognostic indicators in paediatric cerebral malaria: a study of 131 comatose Malawian children [see comments]. *Q J Med* 1989;**71**(265)**:**441-59.

8. Carter JA, Lees JA, Gona JK, et al. Severe falciparum malaria and acquired childhood language disorder. *Dev Med Child Neurol* 2006;**48**(1)**:**51-7.

9. Carter JA, Mung'ala-Odera V, Neville BG, et al. Persistent neurocognitive impairments associated with severe falciparum malaria in Kenyan children. *J Neurol Neurosurg Psychiatry* 2005;**76**(4)**:**476-81.

10. Carter JA, Neville BG, White S, et al. Increased prevalence of epilepsy associated with severe falciparum malaria in children. *Epilepsia* 2004;**45**(8)**:**978-81.

11. Boivin MJ, Bangirana P, Byarugaba J, et al. Cognitive impairment after cerebral malaria in children: a prospective study. *Pediatrics* 2007;**119**(2)**:**e360-6.

12. Ngoungou EB, Poudiougou B, Dulac O, et al. [Persistent neurological sequelae due to cerebral malaria in a cohort of children from Mali]. *Rev Neurol (Paris)* 2007;**163**(5)**:**583-8.

13. Ngoungou EB, Dulac O, Poudiougou B, et al. Epilepsy as a consequence of cerebral malaria in area in which malaria is endemic in Mali, West Africa. *Epilepsia* 2006;**47**(5)**:**873-9.

14. Ngoungou EB, Koko J, Druet-Cabanac M, et al. Cerebral malaria and sequelar epilepsy: first matched case-control study in Gabon. *Epilepsia* 2006;**47**(12)**:**2147-53.

15. Idro R, Carter JA, Fegan G, Neville BG, Newton CR. Risk factors for persisting neurological and cognitive impairments following cerebral malaria. *Arch Dis Child* 2006;**91**(2)**:**142-8.

16. Idro R, Kakooza-Mwesige A, Balyejjussa S, et al. Severe neurological sequelae and behaviour problems after cerebral malaria in Ugandan children. *BMC Res Notes*;**3:**104.

17. John CC, Bangirana P, Byarugaba J, et al. Cerebral malaria in children is associated with long-term cognitive impairment. *Pediatrics* 2008;**122**(1)**:**e92-9.
